# Scrutinizing the Application of Saline Endophyte to Enhance Salt Tolerance in Rice and Maize Plants

**DOI:** 10.3389/fpls.2021.770084

**Published:** 2022-02-17

**Authors:** Zamin Shaheed Siddiqui, Xiangying Wei, Muhammad Umar, Zainul Abideen, Faisal Zulfiqar, Jianjun Chen, Asma Hanif, Shahnaz Dawar, Daniel Anthony Dias, Roomana Yasmeen

**Affiliations:** ^1^Stress Physiology Phenomics Centre, Department of Botany, University of Karachi, Karachi, Pakistan; ^2^Institute of Oceanography, Minjiang University, Fuzhou, China; ^3^Muhammad Ajmal Khan Institute of Sustainable Halophyte Utilization, University of Karachi, Karachi, Pakistan; ^4^Department of Horticultural Sciences, Faculty of Agriculture and Environment, The Islamia University of Bahawalpur, Bahawalpur, Pakistan; ^5^Environmental Horticulture Department and Mid-Florida Research and Education Center, IFAS, University of Florida, Apopka, FL, United States; ^6^School of Health and Biomedical Sciences, Discipline of Laboratory Medicine, RMIT University, Melbourne, VIC, Australia

**Keywords:** endophytes, *Aspergillus terreus*, growth regulator, antioxidant enzyme activity, physiological performance, salinity, crop salt resistance

## Abstract

The present study aimed to witness the plant–microbe interaction associated with salt tolerance in crops. We isolated the endophytic microbe from the root zone of halophytic grass. Later, the salt tolerance of the endophyte was tested in the saline medium and was identified using nucleotide sequencing (GenBank under the accession numbers: SUB9030920 AH1_AHK_ITS1 MW570850: SUB9030920 AH1_AHK_ITS4 MW570851). Rice and maize seeds were coated with identified endophyte *Aspergillus terreus* and were sown in separate plastic pots. Later 21-day-old seedlings were subjected to three NaCl concentrations, including 50, 100, and 150 mM salt stress. Under saline conditions, *A. terreus* showed a substantial increase in growth, biomass, relative water content, oxidative balance, and photochemical efficiency of rice and maize plants. The data reflected that the stimulation of gibberellic acid (GA) in treated leaves may be the main reason for the upregulation of photosynthesis and the antioxidant defense cascade. The data also depict the downregulation of oxidative damage markers malondialdehyde, hydrogen peroxide in rice and maize plants. Conclusively, salt-tolerant endophytic fungus *A. terreus* explicitly displayed the positive plant–microbe interaction by developing salt tolerance in rice and maize plants. Salt tolerance by endophytic fungus coincides with the enhanced GA concentration, which illustrated the stimulated physiological mechanism and gene in response to the extreme environmental crisis, resulting in improved crop productivity.

## Introduction

Exposure to extreme climatic episodes is a serious problem for plant survival in arid or semi-arid regions worldwide. In the last several decades, these drastic changes in the environment and increases in the population have negatively impacted agricultural productivity. Pitman and Lauchli (2002) reported that crop loss due to salinity is challenging to measure, but it is anticipated to increase by 2050. Similarly, abiotic stress, such as salinity and drought, exacerbates these conditions and has been shown to substantially decline agricultural production (Tavakkoli et al., 2011; Abideen et al., 2020). Among the abiotic stress, salinity has affected nearly 1,128 million hectares of land (Wicke et al., 2011). It has previously been reported that crop plants at all stages of their life are sensitive to arid and saline areas, reducing growth (Nawaz et al., 2010). For example, monocot crops such as maize (*Zea mays* L.) and rice (*Oryza sativa* L.) can be considered as the most sensitive or moderately sensitive crops for suboptimal dry saline lands (Shafi et al., 2013). It is known that long-term saline irrigation causes adverse effects on plant physiology and the production of plant metabolites that ultimately affect the total biomass production (Hussain et al., 2019).

In the last few decades, scientists have been searching for sustainable solutions to mitigate salt-related consequences in crops to enhance yield. Several studies have recently suggested alternative and innovative ideas protect plants from the adverse effects of salinity stress (Yu et al., 2019). One strategy is to explore endophytic fungi, which establish a symbiotic relationship with the host plant by providing plant pathogen preventions and synthesis of nutrients that offer favorable conditions to resist sodium chloride (NaCl) toxicity in plants (Afzal et al., 2013; Yu et al., 2019). Physiologically, reactive oxygen species (ROS) increase under salt stress (Majeed et al., 2010; Umar and Siddiqui, 2018). Studies have shown that endophytic fungi can produce biologically active and structurally diverse natural-for-plant defense systems against environmental stresses (Schulz, 2002; Bilal et al., 2019; Yu et al., 2019).

Improving agricultural technology is the only solution to overcome the food demand in a saline environment. Sustainable management practices, genetic engineering, traditional breeding, biofertilizers, and micro-organisms are being used to reduce the saline impact in important crop plants (Tscharntke et al., 2012; Cho et al., 2014; Kamkar, 2016; Yasmeen and Siddiqui, 2018). Planting salt-tolerant crop cultivars had limited success despite significant efforts. Studies suggested that soil micro-organisms are better for increasing soil fertility and crop yield under a saline environment (Vejan et al., 2016; Yasmeen and Siddiqui, 2018). Endophytic fungi are the organisms inhabited in the rhizosphere plants and cultured from almost all crop and wild plants (Ananda and Sridhar, 2002; Wolf and Rashid, 2008). The roots of halophytes also make a symbiotic relationship with fungi. These fungi are considered to be salt tolerant as they are present in saline habitats. It has previously been reported that some fungi play a crucial role in enhancing abiotic stress tolerance in plants (Waqas et al., 2012; Bilal et al., 2019; Tisarum et al., 2020), but their ability to tolerate salt stress in maize and rice is limited. It was suggested that endophytic fungi of saline habitat could enhance salt tolerance, likely due to the symbiotic relationship with the host plant by optimizing water and improving physiological performance under stress conditions. However, little information exists regarding the fungal endophyte ecological, physiological behavior, and growth responses to alleviate salt stress in most essential food crops such as rice and maize (Tisarum et al., 2020).

Rice and maize are the most important cereal crops widely cultivated after wheat (Farooq et al., 2015). The world production of maize has reached 1.15 billion tons and 782 million tons for rice. Rice and maize are grown in a wide range of soil and extreme climatic conditions. Both are important crop plants in the Poaceae family which is moderately sensitive to salt stress (Chinnusamy et al., 2005). Furthermore, broad intraspecific genetic variation for salt resistance exists in rice and maize (Mansour et al., 2005).

A halophyte is a group of plants naturally living in a saline habitat that show diverse growth responses to increasing salinity, ranging from inhibition to dramatic stimulation. All halophytes display a range of salt tolerance with a common feature regulating cellular Na^+^, Cl^–^, and K^+^ concentrations to adjust external water potential. The genus *Aspergillus* is a filamentous endophytic fungus, which is common in soil and plants (Yoo et al., 2018). Due to its minimal nutritional requirements, this fungus can grow on organic and even inorganic materials contaminated by trace organic compounds. It was previously observed that biocontrol agents, i.e., *A. terreus* promoted plant growth (Waqas et al., 2015; Halo et al., 2018). Endophytes living in the plant’s rhizosphere living in a saline habitat could provide natural remedies and establish salt tolerance in crop plants. The modulation of physiological and biochemical cascade owing to the stress tolerance by the applied endophyte (*A. terreus*) was studied in the present research. The current study exploited the information obtained from the physiological and biochemical attributes that witness the microbes (endophyte)-associated metabolic modification, which spells out the novelty of the current approach. The study also enhanced the level of understanding regarding the photochemical attributes during the induction of salt tolerance by endophytes. Therefore, the aim of the present study not only was to isolate and identify the endophyte from the saline habitat but also to scrutinize the effect of salt-tolerant endophytes to mitigate salt stress in two different crop plants belonging to the same family. Furthermore, this study also provides comparative physiological and biochemical details in this regard. It was assumed that endophytes from the halophyte rhizosphere may be more salt resistant and it was checked by *in vitro* experiment.

## Materials and Methods

### Seed Selection

Rice (*Oryza sativa* L.) var. Kernel and maize (*Zea mays* L.) var. NT6621 was obtained in August 2017, from the Plant Protection Department, Karachi, Pakistan. Forty seeds of each rice and maize were surface sterilized in 100 ml of 10% concentrated sodium hypochlorite solution in a beaker for 3 min and washed meticulously by using autoclaved distilled water (100 ml) three times before the beginning of the experiment.

#### Culture Collection and Their Purification

**FIGURE 1:**
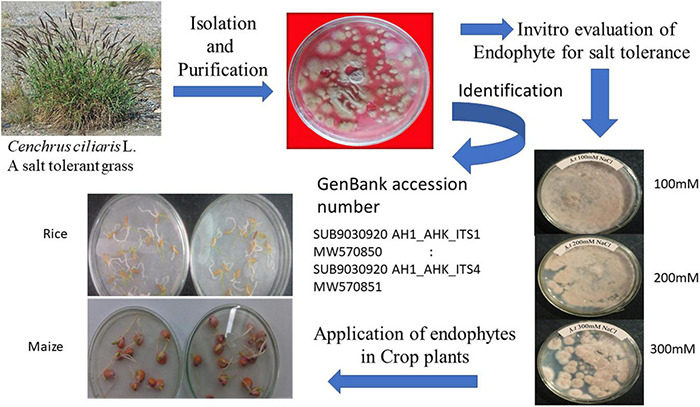
*Aspergillus terreus* was cultured in potatoes dextrose agar containing up to 300 mM NaCl.


The *Aspergillus terreus* was collected from the *Cenchrus ciliaris* L. root zone and was later purified in the Botany Department, University of Karachi (Figure 1). The soil samples were transferred to the laboratory in plastic bags, placed on moist Whatman’s filter paper (sterile) in Petri-plates, and then kept in a humid chamber at 28-30°C for 7 days. The soil dilution plate method was used for isolation and purification. In this regard, the soil was suspended in sterilized water, making a 10% suspension, maintained through agitation using a shaker for 5 min. Later, a series of dilutions (1/10, 1/100, 1/1,000….) was prepared using the suspension until the desired final dilution was achieved. Finally, a dilution factor of 10^4^ to 10^6^ was considered suitable for isolating fungi and bacteria. Aliquots of the final zoospore suspension were prepared by using the dilution method as previously described by Shelford et al. (2005, 2006) using the following equation.

Cells/ml=(AverageNo.ofCells/Square)×(25)×(10)4×(DilutionFactor)

Potato dextrose agar (PDA) was used to culture the fungus on petri-dishes at 28°C for 7 days and checked every 2 days for the emergence of endophytic fungi. The pH of the PDA was maintained to 5.6 before inoculation. The conidia were sub-cultured in a 500-ml flask containing 100 ml of the medium containing 30 g glucose, 3 g yeast extract, 0.39 g KH_2_PO_4_, 1.42 g Na_2_HPO_4_.12H_2_O, 0.060 g MgSO_4_.7H_2_O, 0.70 g NH_4_NO_3_, and 1.0 g KCl per liter (Fargues et al., 1992). *A. terreus* culture was prepared using PDA containing 100, 200, and 300 mM NaCl to examine the salt tolerance capacity (Figure 1). The isolate of *A. terreus* at 300 mM NaCl was incubated at 28-30°C for 24-98 h for further experiments. Subsequently, both rice and maize seeds were coated with *A. terreus* using 2% gum-Arabic adhesive. The colony-forming unit was maintained at 46.3 × 10^–6^ conidia/mL of *A. terreus.*

#### Isolation and DNA Extraction From *Aspergillus terreus*

To identify the *A. terreus*, total genomic DNA was extracted from the mycelium (Lee and Taylor, 1990). The internal transcribed spacer (ITS) regions were amplified using the primer pairs (White et al., 1990; Hong et al., 2005). The temperature profile for the ITS was an initial denaturation step at 95°C (10 min), 35 cycles of denaturation for 30 s at 95°C, annealing at 55°C (30 s) and extension at 72°C (90 s), and final extension step of 10 min at 72°C. The sequencing and purification of PCR products were carried out at Macrogen, Korea. Finally, sequences generated from the analysis were submitted in GenBank under the accession numbers: SUB9030920 AH1_AHK_ITS1 MW570850: SUB9030920 AH1_AHK_ITS4 MW570851.

#### Pot Experiments

Rice and maize seeds were sown in separate plastic pots; each treatment had four pots or replicates (*n* = 4) filled with 500 g of sterile sandy loam soil (9% clay,17% silt, and 74% sand). The experiment was conducted at the Stress Physiology Phenomic Centre, Department of Botany, at the University of Karachi at ambient environmental conditions. The experiments outlined in this research were carried out in a randomized design to assess the impact of the endophytic fungus on host plants. Before the sowing of seeds, the soil was autoclaved to avoid any microbial contamination. Before the experiment, 4.10% soil organic carbon and 0.83% total nitrogen were recorded. For soil organic content measurement, 1 g of soil sample was transferred into a 250-ml conical flask having 10 ml of each 1 N potassium dichromate and concentrated H_2_SO_4_. After 30 min, 50 ml of deionized water, 3 ml of concentrated H_3_PO_4_, and 0.5 ml of 1% diphenylamine indicator were added. Then, titrated slowly with 1 N FeSO_4_ solution up to a green color was achieved. Total nitrite and nitrate-N were determined by acid digestion. For the estimation, the extracted supernatant is reacted with sulfanilic acid and methyl anthranilate as coupling agents to form an azo dye, which is measured at 493 nm using Shimadzu UV–Vis spectrophotometer (pH 7.6 and EC 1.7 dS/m) were recorded using a soil sensor (SDI-12 hydra probe II, Stevens Water, United States) before the beginning of the experiment. For salinity treatments, 150, 100, and 50 mM NaCl concentrations were applied in gradual increments, which are almost equal to 14.5, 9.7, and 4.4 dS/m. A NaCl solution was applied to 21-day-old seedlings using 25 mM NaCl each day, and soil moisture was maintained regularly. Three NaCl levels, including 50, 100, and 150 mM, were implemented by gradual increment, that is, 25 mM NaCl per day, until the desired concentration was achieved. After that, more than 70% soil moisture content was maintained using 60 ± 5 ml distilled water in respective pots on alternate days. Soil moisture contents and electrical conductivity were measured using the soil sensor SDI-12 hydra probe II. Plants without *A. terreus* treatments were rinsed with distilled water and served as control plants. Each experimental setup was exposed under an average day–night temperature (25 ± 4°C and 15 ± 3°C), respectively. Root, shoot length, biomass, physiological parameters, and antioxidant enzymes activities were determined.

### Harvest and Growth Parameters

After 2 weeks of salt exposure, plants were harvested, and growth parameters, such as root, shoot length, and biomass, were determined. Plant material was dried in an oven at 80°C for 24 h. The fresh leaves of each plant were immediately frozen in liquid nitrogen and stored at -20°C for further biochemical analyses.

### Extraction and Estimation of Total Pigments

Five hundred milligrams of fresh leaves were ground in 10 ml of 96% methanol (Batch # 322415 Sigma–Aldrich) and then centrifuged at 4,000 rpm for 10 min. Chlorophyll *a* (C_a_), chlorophyll *b* (C_b_), total chlorophyll [Chl_(a+b)_], and total carotenoids (C_x+c_) were determined according to the method previously outlined in Lichtenthaler (1987). The supernatant (2 ml) was collected, and the absorbance was read at 666, 653, and 470 nm on the spectrophotometer, respectively (UV-1100, Roctec, China). The levels of these pigments were calculated according to the following formulas.


C=a15.65A-6667.340A653

C=b27.05A-65311.21A666

C=x+c1000A-4702.860C-a129.2C/b245

Where, *C*_*a*_ = Chlorophyll a, *C_*b*_* = Chlorophyll b, *Chl*_(a+b)_ = total chlorophyll, *C*_*x+c*_ = Total carotenoids.

### Quantum Yield Photochemical Efficiency and Stomatal Conductance

The measurement of chlorophyll fluorescence emission from 20 randomly selected young leaves of each rice and maize were monitored using a chlorophyll fluorescence meter, model OS-30p^+^ (Opti-Science, United States), before harvesting. A leaf, adapted to dark conditions for 30 min using leaf clips, was initially exposed to the modulated measuring beam of far-red light (LED source with a typical peak at wavelength 735 nm). The original (F_o_) and maximum (F_m_) fluorescence yields were measured under a weak modulated red light (0.5 μmol/m^2^/s) with 1.6 s pulses of saturating light (6.8 μmol/m^2^/s PAR). The variable fluorescence yield (F_v_) was calculated using the equation F_m_–F_o_. The ratio of the variable to maximum fluorescence (F_v_/F_m_) was calculated as the dark-adapted quantum yield of photochemical efficiency photochemistry, and performance index and non-photochemical quenching were calculated as previously described by Maxwell and Johnson (2000). Similarly, stomatal conductance of 20 randomly selected leaves of each treated and control plants of rice and maize were examined by taking the middle portion on the lower surface of the leaves using a leaf porometer (Model SC-1, Decagon).

### Free Proline Determination

The proline content was assessed according to the methods previously described by Bates et al. (1973). Leaf samples (0.5 g) were homogenized in 5 ml sulfosalicylic acid (3% *w/v*) and the homogenate was then filtered using a Whatman #2 filter paper. Subsequently, 2 ml of extract, 2 ml of glacial acetic acid, and 2 ml of acid ninhydrin (1.25 g ninhydrin in 30 ml concentrated glacial acetic acid and 20 mL 6 M phosphoric acid) were added. They were heated in a boiling water bath at 100°C for 1 h. As soon as an observable brick red color developed, the reaction mixture was cooled. Four milliliters of toluene were mixed vigorously with a test tube stirrer for 15–20 s to extract the reaction mixture. The toluene with chromophores was subsequently separated, and absorbance was recorded at 520 nm against the toluene blank. The proline contents were calculated using the following formula:


μmolprolinegFW-1=(μgproline/ml×mltoluene/115.5μg/μmole)/(gsample)/5

### Hydrogen Peroxide Content

Change in hydrogen peroxide content was measured according to the method previously described by Velikova et al. (2000). A 100 mg of freshly harvested leaf samples were homogenized in 3 ml of 0.1% (w/v) trichloroacetic acid in a chilled water bath and then centrifuged at 12,000 g for 15 min. Later, 0.5 ml of 10 mM phosphate buffer (pH 7.0) and 1 ml of 1 M potassium iodide (KI) were added to 0.5 ml of the supernatant. The change in the absorbance of the supernatant was read at 390 nm.

### Leaf Relative Water Content

Randomly, four-leaf strips of 4 × 2 cm^2^ of rice and 1.2 cm^2^ of maize from the mid-veins and the edge were excised, and fresh weights (FW) were determined. For turgid weight (TW), leaves were left in an airtight 90-mm plastic Petri plate filled with distilled water for 12 h. Samples were then dried at 80°C for 48 h in the oven and determined dry weight (DW). The relative water content was determined using the method previously described by Barrs and Weatherley (1962) calculated using the following formula:


Relativewatercontent%=(FW-DW/TW-DW)×100

### Malondialdehyde Content

Measurements of lipid peroxidation in the leaf tissue were determined as the amount of malondialdehyde (MDA) produced by the thiobarbituric acid (TBA) reaction (Dhindsa et al., 1981). Fresh leaves (0.25 g) were homogenized with 0.1% trichloroacetic acid (TCA) in a pestle and mortar and then centrifuged at 10,000 × g for 5 min. One milliliter supernatant was taken, and 4 ml of 20% TCA containing 0.5% TBA was subsequently added. This mixture was heated (95°C) for 30 min in a water bath and immediately cooled. The absorbance of the clear solution was read at 532 and 600 nm. The concentration of MDA was calculated using its extinction coefficient of 155/mM/cm.

### Enzyme Assays

Five hundred milligrams of leaves were crushed and homogenized in prechilled pestle and mortar in 10 ml of protein extraction buffer containing Tris-HCl pH 6.8, 50 mg polyvinylpyrrolidone, and 0.05 mM ethylenediaminetetraacetic acid (EDTA). The homogenate was centrifuged at 12,000 rpm for 10 min using a Smart R-17, Hanil centrifuge machine. The supernatant (5 ml) was collected and used to determine the activities of catalase (CAT), ascorbate peroxidase (APX), and superoxide dismutase (SOD). The total protein was measured using the Bradford assay as previously described by Bradford (1976).

### Assay of Catalase Activity

Catalase (EC 1.11.1.6) activity was estimated using the method previously described by Patterson et al. (1984). The decomposition of H_2_O_2_ was measured at 240 nm taking Δε as 43.6/mM/cm. The reaction mixture (3.0 ml) consisted of 10.5 mM H_2_O_2_ in 0.05 M potassium phosphate buffer (pH 7.0), which was initiated after adding 0.1 ml of the enzyme extract at 25°C. The decrease in absorbance at 240 nm was used to calculate the activity. One unit of CAT activity is defined as the amount of enzyme that catalyzes the conversion of 1 mM of H_2_O_2_/min at 25°C.

#### Assay of Ascorbate Peroxidase Activity

Ascorbate peroxidase (EC 1.11.1.11) activity was carried out using the method previously described by Nakano and Asada (1981). The reaction mixture (2.0 mL) contained 50 mM potassium phosphate buffer (pH 7.0), 0.2 mM EDTA, 0.5 mM ascorbic acid, and 0.25 mM H_2_O_2_. The reaction was initiated after the addition of 0.1 ml of the enzyme extract at 25°C. The decrease in absorbance at 290 nm for 1 min was recorded and the amount of ascorbate oxidized was calculated from the extinction coefficient 2.8 mM/cm. The unit of activity was expressed as a micromole of ascorbic acid oxidized per minute at 25°C.

#### Assay of Superoxide Dismutase Activity

Superoxide dismutase (EC 1.15.1.1) activity was carried out according to the method previously described by Beyer and Fridovich (1987). The reaction mixture consisted of 27 ml of 0.05 M potassium phosphate buffer (pH 7.8), 1.5 ml of L-methionine (300 mg per 2.7 ml), 1.0 ml of nitroblue tetrazolium salt (14.4 mg per 10 ml), and 0.75 ml of Triton X-100. Aliquots (1.0 ml) of this mixture were transferred into glass tubes, followed by adding 20 ml of the enzyme extract and 10 ml of riboflavin (4.4 mg per 100 ml). The reaction mixture was mixed and then illuminated for 15 min in an aluminum foil-lined box containing 25 W fluorescent tubes. The sample was substituted with 20 ml of buffer in the control tube, and the absorbance was measured at 560 nm. The reaction was quenched by switching off the light and placing the tubes in the dark. An increase in the absorbance due to the formation of formazan was measured at 560 nm. Under the described conditions, the increase in absorbance in the control was taken as 100% and the enzyme activity in the samples was calculated by determining the percentage inhibition per minute. One unit of SOD was the amount of enzyme that causes a 50% inhibition of the rate for reduction of the nitroblue tetrazolium salt under the conditions of the assay.

#### Assay of Guaiacol Peroxidase Activity

Guaiacol peroxidase (GPX; EC 1.11.17) activity was measured spectrophotometrically at 25°C using the method previously described by Tatiana et al. (1999). The reaction mixture (2.0 ml) consisted of 0.05 M potassium phosphate buffer (pH 7.0), 2 mM H_2_O_2_, and 2.7 mM guaiacol. The reaction was initiated by the addition of 0.1 ml of the enzyme extract. The initial rate of guaiacol oxidation was measured by the formation rate of tetra guaiacol and was measured at 470 nm (Δε = 26.6 mM/cm). One unit was defined as the amount of enzyme required to catalyze one micromole of hydrogen peroxide conversion, with guaiacol as hydrogen donor, per minute under specified conditions.

### Extraction and Estimation of GA_3_

Leaves from each treatment and control were extracted using ethyl acetate three times (50 + 25 + 25) using Rachev et al. (1993). Subsequently, the extract was separated using anhydrous sodium sulfate (5 g) and evaporated under reduced pressure at 45°C at 15 rpm. The residual extract was re-dissolved in HPLC grade acetonitrile and kept for subsequent thin layer chromatography (TLC) analysis. TLC’s quantitative estimation of gibberellins was carried out using standard gibberellins (Cavell et al., 1967). Benzene: *n*-butanol:acetic acid (6:3:1) was used as the mobile phase and the developed spots were visualized under UV (254 nm) after spraying with a mixture of ethanol:concentrated sulfuric acid in a (95:5) ratio.

### Determination of Na^+^, K^+^, and Cl^–^ Ions

Sodium, potassium, and chloride ions were determined in leaf tissues (Chaudhary et al., 1996). Five hundred milligrams of randomly collected dry leaves were homogenized in 10 ml of double-distilled water at 25°C for 10 min. The mixture was centrifuged for 15 min at 3,000 × *g* and the supernatant filtered through Whatman no.1 filter paper. Subsequently, the filtrate was used to estimate Cl^–^ by titration with the AgNO_3_ solution using 5% K_2_CrO_4_ as an indicator, whereas Na^+^ and K^+^ by flame photometer (Jenway, PFP-7).

### Statistical Analyses

**FIGURE 2:**
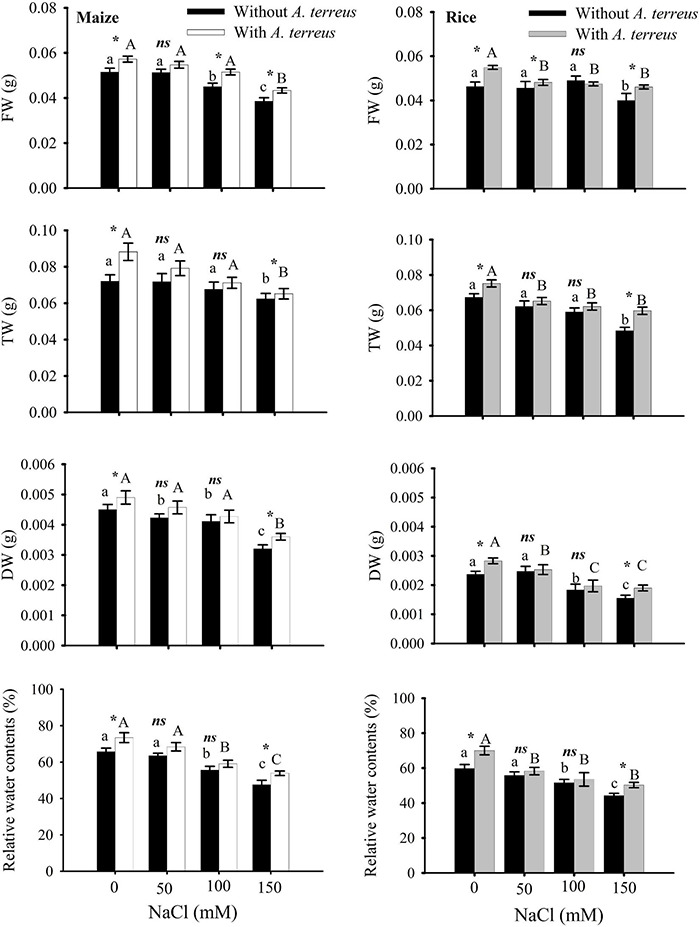
Effect of *Aspergillus terreus* seed treatments on relative water content and biomass accumulation of maize (*Zea mays* L.) var. NT6621 and rice (*Oryza sativa* L.) var. The kernel is in a saline environment. Vertical lines on bar graphs denote mean standard error (±). Same letters on bar graphs denote non-significant differences within a treatment (capital letters for *A. terreus*, small letters without *A. terreus* only salinity), (*) denotes significant differences, and (*ns*) stands for non-significant among the treatments (with and without *A. terreus*).

**FIGURE 3:**
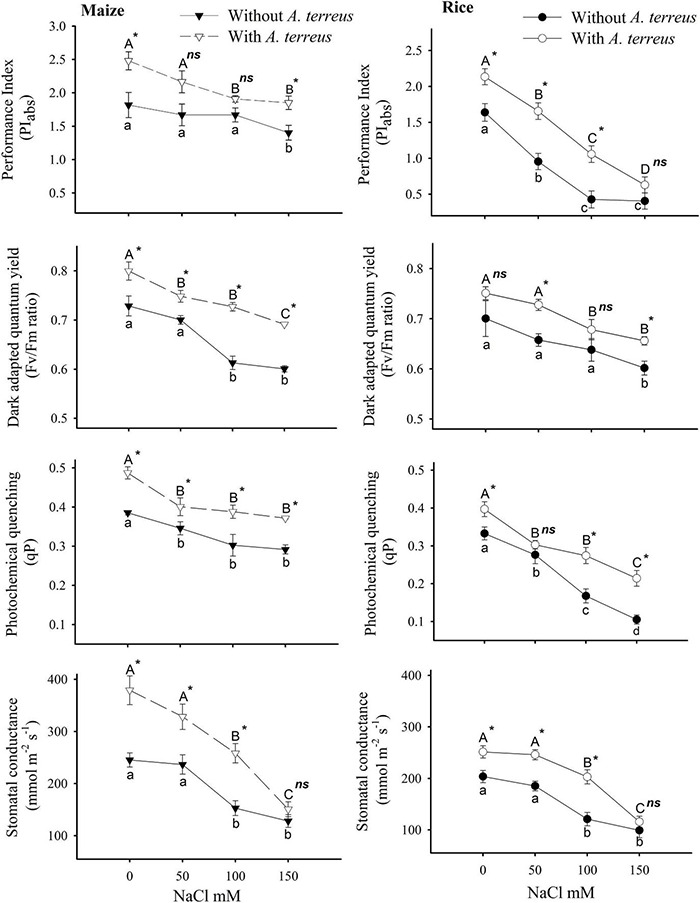
The effect of *Aspergillus terreus* seed treatments on the photosynthetic attributes of maize (*Zea mays* L.) var. NT6621 and rice (*Oryza sativa* L.) var. Kernel under saline conditions. Vertical lines on line graphs denote mean (±) standard error. Same letters on the bar graphs denote non-significant differences within a treatment (capital letters for with *A. terreus*, small letters without *A. terreus* only salinity), (*) denotes significant differences, and (*ns*) denotes non-significant among treatments (with and without *A. terreus*).

**FIGURE 4:**
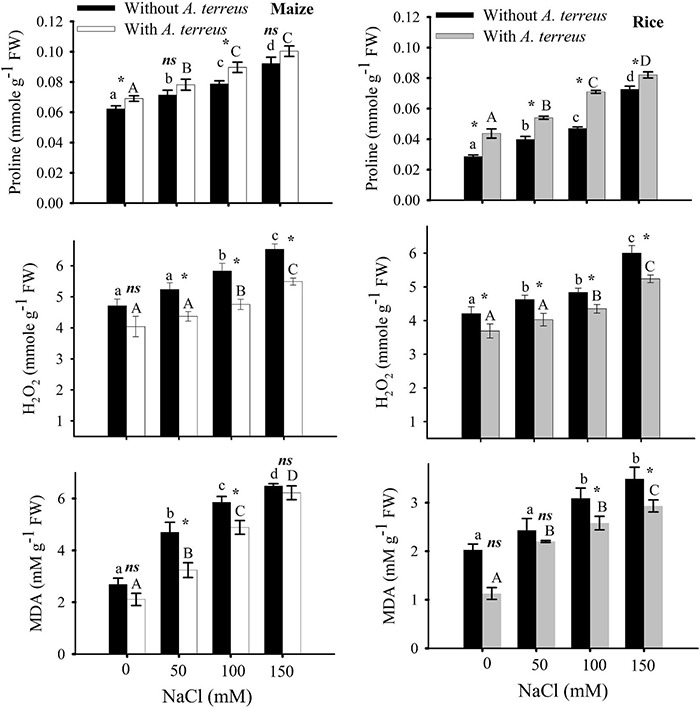
Effect of *Aspergillus terreus* seed treatments on the proline, H_2_O_2_, and MDA contents of maize (*Zea mays* L.) var. NT6621 and rice (*Oryza sativa* L.) var. Kernel under saline conditions. Vertical lines on bargraphs denote mean (±) standard error. The same letters on the bar graph denote non-significant differences within a treatment (capital letters for *A. terreus*, small letters for without *A. terreus* only salinity), (*) denotes significant differences, and (*ns*) denotes non-significant among treatments (with and without *A. terreus*).

**FIGURE 5:**
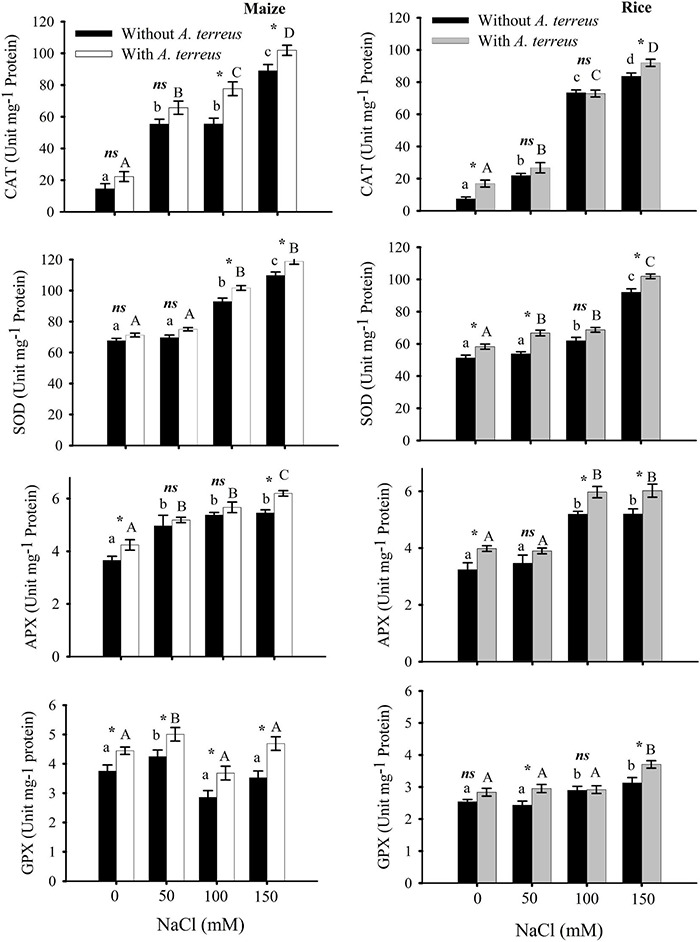
The effect of *Aspergillus terreus* seed treatments on antioxidants enzymes of maize (*Zea mays* L.) var. NT6621 and rice (*Oryza sativa* L.) var. Kernel under saline conditions. Vertical lines on bar graphs denote mean (±) standard error. Same letters on the bar graph denote non-significant differences within a treatment (capital letters for *A. terreus*, small letters without *A. terreus* only salinity), (*) denotes significant differences, and (*ns*) denotes non-significant among treatments (with and without *A. terreus*).

**FIGURE 6:**
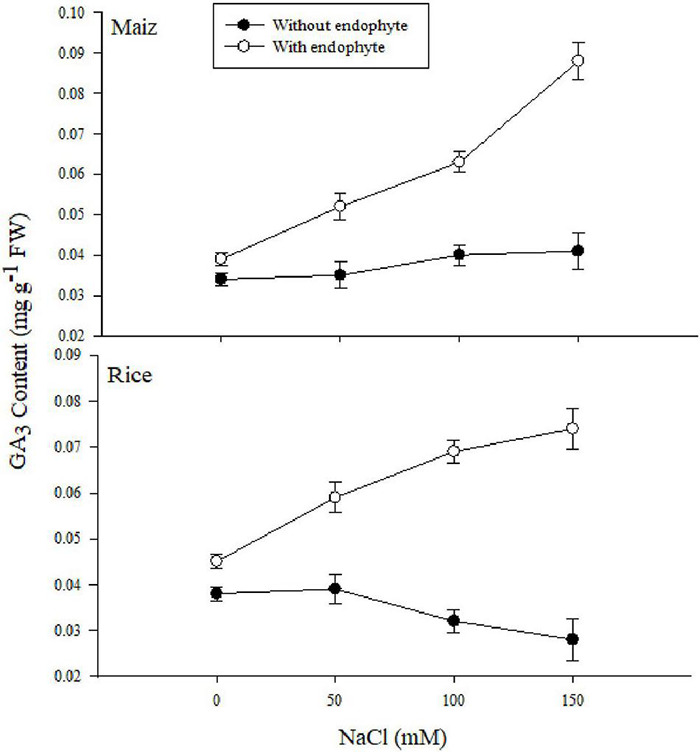
The effect of *Aspergillus terreus* seed treatments on total gibberellic acid of maize (*Zea mays* L.) var. NT6621 and rice (*Oryza sativa* L.) var. Kernel under saline conditions. Vertical line online graph represents mean SE (±).

The data generated from the treated and control groups (*n* = 4) were subjected to statistical analyses using the software SPSS Version 20 (IBM, United States). A two-way ANOVA was used to determine significant differences among means within and among each treatment. The significance of the applied Bonferroni test has been represented on bar graphs (Figures 2–6). Same letters denoted on bar graphs show non-significant differences (*p* < 0.05) within a treatment (Capital letters for *A. terreus*, small letters without *A. terreus*, only salinity), (*) denotes significant differences, and (*ns*) denotes non-significant among the treatments (with and without *P*).

## Results

The effects of *A. terreus* with and without salt stress on maize and rice plants were examined in this experiment. Growth in root and shoot length declined significantly in salt stress (NaCl) treatments as compared to positive control. However, seeds treated with *A. terreus* showed a significant increase in shoot lengths of maize up to 30% and rice up to 21% under salt stress as compared to untreated plants (Supplementary Table 1). Similarly, the application of *A. terreus* improved the root lengths in both plants (maize 26%, rice 29%) against salt stress. At 150 mM NaCl, a plant treated with *A. terreus* showed up to a 13% increase in rice and a 10% increase in maize fresh weight (FW) as compared to untreated plants (Figure 2). Dry weight (DW) also increased in rice (18%) and maize (10%) at 150 mM when plants were treated with *A. terreus.* Leaf relative water content (RWC) also increased in maize and rice plants under salt stresses when plants were exposed to endophyte *A. terreus*.

It was evident from the data that *A. terreus* mitigates the deleterious effect of NaCl stress in both maize and rice plants improving chlorophyll content under salt stress conditions (Supplementary Table 2). The *A. terreus* in maize plants increased the total chlorophyll up to 24% under salt stress environment. The use of *A. terreus* enhanced chlorophyll ‘a’ and ‘b’ displaying a substantial increase in maize (36%) and rice (38%) at 150 mM NaCl as compared to untreated plants.

However, the application of endophyte substantially improved the performance index (PI_abs_) under salt stress compared to endophyte untreated plants. In both plants, the variable fluorescence (F_v_/F_m_) ratio was minimally affected it was in the range of 0.6–0.7 under salt stress. Photochemical quenching (qP) exhibited more significant variations under salt stress, especially in rice plants. A sharp decline (almost two folds) in qP was observed in rice as the salt concentration increased. Application of endophyte in maize plants expressed better qP values around 0.3 under salt stresses than rice plants, where qP values decreased up to 0.1. These results suggested that maize plants had better photosynthetic performance than rice plants under a salt stress environment. However, the use of *A. terreus* in both plants improved the photosynthetic performance under salt stress conditions (Figure 3).

In stress without *A. terreus*-free proline content was increased in both maize and rice and it was in the range of 0.02–0.0727 (Figure 4). However, the use of endophyte (*A. terreus*) in rice and maize alleviated the adverse effects of salinity and showed substantial increases in proline; almost 9-12% in maize and 11-34% in rice under salt stress conditions. It was noticed from the data that the amount of hydrogen peroxide (H_2_O_2_) was greatly decreased in maize (19%) in the presence of *A. terreus* under saline conditions compared to rice plants. Seeds treated with *A. terreus* showed a minimum H_2_O_2_ (almost 19% in maize and 14% in rice) at all NaCl treatments as compared to *A*. *terreus* untreated plants. MDA content of *A. terreus*-treated plants was decreased in both maize and rice as compared to untreated plants (Figure 4). It appears that the *A. terreus* treatment mitigated the salinity effects and reduced MDA up to 44% in maize and 79% in rice under salt stress environment (Figure 4).

Antioxidant enzymes including CAT, APX, SOD, and guaiacol peroxidase (GPX) activities were measured at different NaCl concentrations with and without *A. terreus* (Figure 5). Observations revealed that both maize and rice plants CAT, APX, and SOD activities substantially increased with an increasing NaCl concentration in the presence of endophyte *A. terreus*. Maize plants expressed 12–28% CAT, 7–9% SOD; 4–12% APX; 15–25% GPX activities. At 50 mM NaCl, the CAT activities of maize plants were two-fold higher than rice plants. In the same way, GPX activities of maize were also greater than rice plants under salt stress. These results showed that maize plants had greater antioxidant activity than rice (Figure 6).

The gibberellic acid (GA) content increased in both maize and rice under salt stress due to the application of *A. terreus* (Figure 6). However, a gradual increase was observed in both rice and maize. Results suggested that maize plants had higher GA under 150 mM NaCl treatments with and without *A. terreus* compared to rice in each treatment.

Salt stress affects photosynthesis and plants’ growth due to ion toxicity and osmotic drought. Under saline conditions, Na^+^, K^+^, and Cl^–^ concentrations were examined in both rice and maize plants (Table 1). Results showed that maize and rice had accumulated substantial amounts of Na + and Cl- ions under NaCl stresses. However, after the use of endophyte *A. terreus*, the Na^+^ accumulation was reduced up to 103% in maize and 73% in rice plants under salt stress. *A. terreus* showed a more significant reduction in Na^+^ and Cl^–^ ions in maize than in rice plants. Plants exposed to salt stress without *A. terreus* showed the highest amounts of Na^+^ and Cl^–^ as compared to plants inoculated with *A. terreus*. Results expressed that rice and maize had lower Na^+^ concentrations as compared to Cl^–^ under salt stress. Similarly in salt stress, a minimal decline in K^+^ ion was also observed in both plants without *A. terreus* (Table 1). The *A. terreus*-inoculated maize plants expressed a lesser decline in K^+^ ion accumulation as compared to rice under salt stress. These outcomes showed that the K^+^ concentration was higher in maize compared to rice under salt stress. The higher concentration of K^+^ in maize than rice might be due to the inoculation of *A. terreus*, which maintains the membrane integrity and averts the leakage of K^+^ under salt stress conditions. The Na^+^/K^+^ ratio help in salt tolerance in plants. The Na^+^/K^+^ ratio was increased under salt stress as compared to control plants. The maize plants have a lower Na^+^/K^+^ ratio as compared to rice plants.

**TABLE 1:** Changes in Na^+^, K^+^, and Cl^–^ concentration (mM) of rice and maize due to *Aspergillus terreus* seed treatments under saline conditions.

Species	NaCl concentration	Without *A. terreus*				With *A. terreus*			
		Na^+^	K^+^	Na^+^/K^+^	Cl^–^	Na^+^	K^+^	Na^+^/K^+^	Cl^–^
**Maize**	0 mM	2.2^a^	6.2^a^	0.35	1.8^a^	1.7^a^	6.4^a^	0.27	1.6^a^
	50 mM	65.2^b^	5.8^b^	11.24	69.2^b^	32.1^b^	6.2^a^	5.18	35.6^b^
	100 mM	87.5^c^	5.9^b^	14.83	106^c^	44.1^c^	5.9^a^	7.47	51.2^c^
	150 mM	101.6^d^	4.5^c^	22.58	115^d^	52.5^d^	5.5^b^	9.55	62.2^d^

**Rice**	0 mM	2.8^a^	5.9^a^	0.47	2.2^a^	1.9^a^	6.1^a^	0.31	1.85^a^
	50 mM	60.1^b^	5.6^a^	10.73	101^d^	35.2^b^	5.9^b^	5.97	39.4^b^
	100 mM	82.5^c^	5.5^a^	15.00	114^c^	56.2^c^	5.7^c^	9.86	48.2^c^
	150 mM	109^d^	4.1^b^	26.59	121^d^	62.8^d^	5.4^d^	11.63	56.1^d^

*Similar alphabets within the salinity treatments represent non-significant at P = 0.01. Different lowercase letters represent significant difference among the treatments.*

## Discussion

Salt-tolerant endophytic fungus *A. terreus* was applied in two different crops, i.e., maize and rice belonging to the same family. The plants’ growth and physiological responses under saline stress conditions were determined. It was observed that salinity caused a substantial reduction in the growth and biomass of those plants without *A. terreus* treatment. The application of *A. terreus* mitigates salt-related consequences in plants, which results in considerable increases in growth, biomass production (up to 30% in maize; 25% in rice), and RWC (11% maize; 14% rice) under saline conditions (Supplementary Table 1).

Endophytic fungus negates the salt stress in plants by activating the antioxidant system, increasing the level of osmoprotectants, modulation of phytohormone profile, and reducing the salt-induced root respiration (Jogawat et al., 2013; Rewald et al., 2015; Ghaffari et al., 2016; Zhang et al., 2019). These cascades of effects are in feedback to enhance the plant growth and resistance against salt stress. The present study observed the increased biomass content in maize and rice plants treated with endophytic fungus. This explicates the modification in the root architecture system, by the endophytic fungus to improve the plant’s nutrient uptake which results in increased accumulation of biomass content (Gupta et al., 2020). Improved root architecture system by endophytic fungus with different compositions of rhizo-deposits (exudates) also enables the rice and maize plants to utilize water resources of the salt-exploited areas until it cannot be avoided (Jogawat et al., 2013). This imparted the effect on the plant water status for regulation of salt acquisitions and translocation with the increased RWC of the plants treated with endophytes (Figure 2). The current study also witnessed the increased root length in rice and maize plants colonized with endophytes which negated the inhibition of cell division and elongation caused by salt acquisitions in the soil (Rahnama et al., 2011). The increased shoot length of the plants colonized with endophytes was due to the phytohormone modulations. Inoculation of endophytes strategizes the interplay of phytohormone to regulate the physiological and metabolic process against salt stress in plants. From Figure 6, it is evident that the increased bioactive gas was responsible for the greater shoot length in maize and rice plants (Waqas et al., 2012). The greater increment in maize as compared to rice plants might be linked with the better symbiotic association of an endophytic fungus with the maize than the rice (Rho et al., 2018).

Higher chlorophyll content (a and b) as well as carotenoids in rice and maize plans colonized with *A. terreus* spell out the induction of salt tolerance in the chloroplast by endophyte inoculation (Supplementary Table 2). Excessive Na + ion accumulation in chloroplast deters the photosynthetic activity, which was evident from the low chlorophyll and carotenoid content of plants without endophyte inoculation (Ali et al., 2012). The current findings suggest that the biosynthetic enzymes of the chlorophyll pathway could be more active and functional in rice and maize plants, which were inoculated with *A. terreus* against salt stress (Chaves et al., 2009). The enhanced level of gs (Figure 3) with the *A. terreus* inoculation depicted the tolerance of osmotic shock and increased leaf area caused by the salt stress (Chaves et al., 2009). Higher gs favor the enhancement of photosynthetic attributes by providing more CO_2_ assimilation and rubisco activity (Seemann and Critchley, 1985). The increased photosynthetic attributes in plants inoculated with endophyte displayed the reduced risk of photosystem II damage, which is often caused by salt stress. The higher performance index (PI_abs_), dark-adapted quantum yield (F_v_/F_m_), and photochemical quenching (qP) of photosynthetic apparatus were due to less accumulation of electrons in the thylakoid membrane and reduced disposal of energy dissipation among the plants colonized with endophytes (Habib et al., 2017). These photochemical attributes displayed the protection of D1 and D2 proteins of photosystem II by *A. terreus* against salt stress.

A decrease in MDA (44% maize; 19% rice) and H_2_O_2_ (22% maize; 14% rice) in *A. terreus*-treated plants under a saline environment was observed. In the present study, plants treated with *A. terreus* had lower H_2_O_2_ contents than *A. terreus*-untreated plants under salinity stress (Figure 3). It was suggested that several plants develop several physiological and biochemical tactics to deal with stress in a stressful environment. This osmotic adjustment substance that exists in plant cells is more important as it is available in the free state and possesses high water solubility, low molecular weight, and helps in maintaining physiological pH. It was observed that plant cells tend to accumulate soluble osmotic substances such as proline, sugars, and sugar-containing alcohol, to alleviate osmotic stress caused by salt stress (Silveira et al., 2003). It was evident from the present study that the application of endophytes lowered the H_2_O_2_ contents indicating the relief of stress-related consequences (Ahmad et al., 2015; Tahjib-Ul-Arif et al., 2018). It is presumed that the application of endophytes minimizes the breakdown of aldehydic lipid products, causing a reduction of MDA and H_2_O_2_ values under saline conditions and mitigating salt stress. In this investigation, *A. terreus*-treated plants expressed lower MDA contents under saline environments. Our findings agreed with a study previously reported by Khan et al. (2012) that showed more than 40% decline in MDA production under saline conditions, which suggested lower cellular membrane damage in plants.

In abiotic stress such as salinity, elevated H_2_O_2_ production damages the protein and lipid molecules (Umar and Siddiqui, 2018). It is presumed that the application of *A. terreus* lowered the H_2_O_2_ production in salt stress due to more antioxidant enzyme activity. Moreover, it was reported that an endophyte such as *Trichoderma* enhances antioxidant enzymes such as glutathione S-transferases (GSTs) and peroxidase (POD) activities and lowers reactive oxygen species (ROS) production (Hajiboland et al., 2010; Wu and Sardo, 2010; Alqarawi et al., 2014). It was reported that maximum quantum yield and photosynthetic performance control the production of ROS (Kalaji et al., 2018). Photosynthetic performance and quantum yield are inter-related and are often decreased in abiotic stress and under elevated ROS levels. Fluctuating responses with respect to quantum yield and performance index in a stress environment are diversified and specific for some plant species (Behera et al., 2002). Substantial photosynthetic performance index and quantum yield in maize treated with *A. terreus* indicate that maize could develop a better symbiotic relationship with *A. terreus* in a saline environment compared to rice. In our study, it was detected that the presence of *A. terreus* in a saline environment increased the activity of antioxidant enzymes as compared to those saline media that did not have *A. terreus*. Earlier, it was reported that the activities of antioxidant enzymes such as catalase (CAT), superoxide dismutase (SOD), peroxidase (POD), and ascorbate peroxidase (APX) were increased in a saline environment (Umar and Siddiqui, 2018). It is presumed that the presence of *A. terreus* in a saline environment diminished H_2_O_2_ production due to elevated antioxidant enzyme activities as well as proline production. It was reported that antioxidant enzymes and the production of osmolytes and polyols, such as proline and sorbitol, are important physiological strategies for coping with the consequences of abiotic stress and maintaining ion homeostasis (Sairam et al., 2002; Rasool et al., 2013). Antioxidant activities are important physiological aspects playing a key role in coping with salt stress (Guo et al., 2018). An increase in antioxidant activities protects the cell against environmental stresses like drought and salt stress. *A. terreus*-treated plants showed significant increases ranging from 5 to 28% in antioxidant enzymes activities, including CAT, APX, SOD, and GPX, compared to non-inoculated plants under salt stresses (Figure 6). It was observed that the endophytic fungi increased antioxidant enzyme activities in plants under salt stress conditions (Rasool et al., 2013; Hashem et al., 2014). Physiologically, abiotic stresses cause oxidative damage to plants by producing ROS in plant cells, which are compensated by provoking an antioxidant system (Ahmad et al., 2015). The production of ROS was elevated in stress conditions causing denaturation of biomolecules (Becana et al., 2000; Majeed et al., 2010). In this regard, antioxidant enzymes such as SOD play a critical role in plants and functionally convert the superoxide radicals into water and oxygen (O_2_). Elevated SOD activity against salinity in the present study seems to be used in the *de novo* synthesis of antioxidant enzymatic proteins such as APX, CAT, and GPX (Verma and Dubey, 2003; Malecka et al., 2007; Majeed et al., 2010). Therefore, it may also be suggested that a combined effect of two or more antioxidant enzymes could provide complete detoxification of ROS to plants under salt stress conditions, as observed in rice and maize.

Plants treated with *A. terreus* had lower concentrations of Na^+^ and Cl^–^ under salt stress than control plants favoring a better plant growth and photosynthetic performance. Results suggested that *A. terreus* improved the efficiency of plants to eliminate the Na^+^ and Cl^–^ under salt stress. Both plants treated with *A. terreus* showed a greater concentration of K^+^ (3–24% increment), whereas lower Na^+^ concentrations (46–98% decline) compared to plants without *A. terreus* under salt stress. The increment in K^+^ suggests that *A. terreus* had greater potential to accumulate K^+^ ions under NaCl playing an important role in maintaining physiological processes under salt stress (Colpan et al., 2013). Furthermore, K^+^ ion minimizes oxidative stress and improves the root and shoot length (Jan et al., 2017). The essential criteria for salt tolerance in plants are higher amounts of K^+^ and Na^+^ selectivity by roots and distribution of ions in plant tissues under salt stress (Gu et al., 2016). The higher the accumulation of Na^+^ and Cl^–^ ions are frequently assumed to be primarily responsible for reducing photosynthesis and growth of plants (Tsai et al., 2004; Hong et al., 2009).

Application of endophyte showed a substantial decrease in MDA. The higher K^+^ contents in the endophyte-treated plant are linked with reduced MDA contents in monocot plants (Jan et al., 2017). It is suggested that the *A. terreus*-treated plant showed higher K + contents that ultimately prevent membrane degradation in maize and rice plants under salt stress. Na^+^/K^+^ ratio under salt stress is an important trait to screen the genotypes. It was interesting that maize and rice plants inoculated with *A. terreus* showed a lower Na^+^/K^+^ ratio than uninoculated plants under salt stress. This indicated that the Na^+^ exclusion mechanism might be more active in *A. terreus*-inoculated plants. The salt tolerance in *A. terreus*-inoculated plants might be due to the lower rate of Na^+^ loading and better capacity to sequester as it enters the leaf (Alias et al., 2020). Fungus-treated plants, lower Na^+^/K^+^ ratio in maize plants under salt stress may have helped in better tolerance than rice plants.

Some of the vital stress parameters were selected and compared using Pearson’s correlations. Supplementary Table 3 explains the correlation analysis with and without *A. terreus* among the desired parameters. Proline, CAT, APX, and GPX were positively correlated with other RWC, Chl, PI_abs_, F_v_/F_m_, and stomatal conductance (gs), and RWC showed the highest negative correlation (–0.990^**^ and –0.950^**^) with H_2_O_2_ and MDA under salt stress treatment. It was evident from the data that proline accumulation could be the main reason for higher RWC in plants treated with endophyte under saline habitat. Similarly, higher antioxidant activities also provide further evidence in this regard. It was suggested that an increased MDA depended on lower RWC under stress conditions. Furthermore, RWC positively correlated with Chl, PI_abs_, F_v_/F_m_, and gs (almost 0.95^**^), which expressed the importance of RWC in photosynthesis (Umar and Siddiqui, 2018). Higher RWC improves the photosynthetic efficiency by increasing chlorophyll contents and photochemical performance (Hajlaoui et al., 2009; Umar and Siddiqui, 2018). The correlation analysis showed that GA_3_ was positively correlated with CAT and SOD. The photosynthetic attributes were also significantly correlated with GA_3_ concentration. This correlation suggested that GA_3_ upregulated the antioxidant enzyme activities linked with better photosynthetic performance in maize and rice plants against *A. terreus* treatment.

The role of GA producing endophytes in crop plants such as maize and rice regarding upregulating physiological traits was not previously highlighted. In the present study, it was evident from the data that *A. terreus* improved substantial plant growth, photosynthetic performance index, and antioxidant system are linked with excessive production of GA in both the treated plants. Furthermore, it was suggested that *A. terreus* improved growth and provided physiological tolerance in crop plants due to more phytohormones (GA_3_) production under saline conditions. However, the concentration of GA production in both plants varies, affecting the physiological and growth response differently under the abiotic stress environment. Our current findings indicated that *A. terreus* established a more significant symbiotic relationship with maize than rice under saline conditions producing phytohormones, showing the possibility of using endophytic microbe in salt-affected soil. This profound eco-physiological benefit suggests that *A. terreus* could be considered a better plant biostimulant for improving maize seedling in saline soil than rice. Our findings also indicated that rice and maize could produce biomass for economic sustainability by using brackish water irrigation on saline soils avoiding competition with conventional agriculture.

## Data Availability Statement

The datasets presented in this study can be found in online repositories. The names of the repository/repositories and accession number(s) can be found below: https://www.ncbi.nlm.nih.gov/genbank/, SUB9030920 AH1_AHK_ITS1 MW570850: SUB9030920 AH1_AHK_ITS4 MW570851.

## Author Contributions

ZS, RY, and MU did physiological and photochemistry experiments. DD did proofreading and editing. AH and SD were involved in providing fungus and its molecular identification. All authors equally participated in the manuscript.

## Conflict of Interest

The authors declare that the research was conducted in the absence of any commercial or financial relationships that could be construed as a potential conflict of interest. The handling editor declared a past co-authorship with one of the authors FZ.

## Publisher’s Note

All claims expressed in this article are solely those of the authors and do not necessarily represent those of their affiliated organizations, or those of the publisher, the editors and the reviewers. Any product that may be evaluated in this article, or claim that may be made by its manufacturer, is not guaranteed or endorsed by the publisher.
