# Supplementary material for: Scrutinizing the Application of Saline Endophyte to Enhance Salt Tolerance in Rice and Maize Plants
Source: Front Plant Sci. 2022 Feb 17;12:770084. doi: 10.3389/fpls.2021.770084 (PMC8891170; doi:10.3389/fpls.2021.770084)
Supplement: Supplementary file 1 [file Table_1.docx]

**Tab. S1**. *Aspergillus terreus* seed treatments showed increases in growth and biomass accumulation in rice and maize under saline conditions. Data is presented in the form of mean ± standard error. Same letters on the data denotes non- significant differences within each treatment (*****) denotes significant and (*ns*) non-significant difference among treatments (with and without *A. terreus*).

|  |  |  | **Without *A. terreus*** | | |  | |  |  | ***With A. terreus*** | | |  | |  |
| --- | --- | --- | --- | --- | --- | --- | --- | --- | --- | --- | --- | --- | --- | --- | --- |
|  |  | **Shoot** | | | **Root** | |  | | **Shoot** | | **Root** | | | |  |
|  |  | **Length (cm)** | **Dry wt (gm)** | **Length (cm)** | | **Dry wt (gm)** | |  | **Length (cm)** | **Dry wt (gm)** | | **Length (cm)** | | **Dry wt (gm)** | |
| **Maize** | 0mM | 28.93±1.31**a*** | 0.19±0.01**a*ns*** | 13.77±2.09**a*** | | 0.14±8.2500e-3**a*** | | | 31.77±1.26**A*** | 0.21±0.01**A*ns*** | | 15.43±2.16**A*** | | 0.187±7.5e-3**A*** | |
|  | 50mM | 23.73±0.68**b*** | 0.13±0.01**b*ns*** | 12.30±2.12**b*** | | 0.14±7.2500e-3**a*** | | | 28.10±1.42**A*** | 0.16±0.01**B*ns*** | | 14.30±2.04**A*** | | 0.186±8.4e-3**A*** | |
|  | 100mM | 13.70±0.61**b*** | 0.10±6.7e-4**b*ns*** | 09.83±1.30**c** | | 0.02±7.6400e-3**b*** | | | 17.30±1.54**B*** | 0.10±2.3e-3**B*ns*** | | 13.30±1.50**B*ns*** | | 0.094±7.5e-3**B*** | |
|  | 150mM | 10.09±1.01**c*** | 0.07±6.7e-3**c** | 09.87±1.06**c*ns*** | | 0.02±8.7600e-3**b*** | | | 14.47±1.43**C*** | 0.08±2.8e-3**B*ns*** | | 10.77±0.88**B*** | | 0.084±5.2e-3**B*** | |
|  |  |  |  |  | |  | |  |  |  | |  | |  | |
| **Rice** | 0mM | 13.96±0.66**a*** | 0.03±0.002**a*** | 10.62±0.31**a*** | | 0.020±1.5e-3**a*ns*** | |  | 16.10±1.08**A*** | 0.040±2.4e-3**A*** | | 13.23±0.19**A*** | | 0.018±1.5e-3**A*ns*** | |
|  | 50mM | 12.43±0.32**b*** | 0.03±0.003**a*** | 10.09±0.32**a*** | | 0.020±1.5e-3**a*ns*** | |  | 14.13±0.40**A*** | 0.040±2.4e-3**A*** | | 11.03±0.32**B*** | | 0.017±1.5e-3**Ans** | |
|  | 100mM | 07.07±0.28**c*** | 0.009±2.2e-4**b*** | 08.00±0.20**b*** | | 0.009±1.2e-4**bns** | |  | 09.50±0.36**A*** | 0.010±2.7e-3**B*** | | 10.39±0.46**B*** | | 0.011±8.5e-4**B*ns*** | |
|  | 150mM | 06.78±0.22**d*** | 0.007±2.2e-5**c*** | 07.91±0.22**c*** | | 0.006±2.3e-4**c*** | |  | 08.65±0.07**B*** | 0.009±4.0e-5**C*** | | 06.11±0.08**C*** | | 9.2e-3±7.0e-5**C*** | |
|  |  |  |  |  | |  | |  |  |  | |  | |  | |

**Table S2**. Changes in the chlorophyll content of both rice and maize due to *Aspergillus terreus* seed treated under saline conditions. Data is presented in the form of the mean ± standard error. Same letters on the data denotes non- significant differences within each treatment (*****) denotes significant and (*ns*) non-significant differences among treatments (with and without *A. terreus*).

|  |  |  | **Without *A. terreus*** | |  |  | **With *A. terreus*** | |  |
| --- | --- | --- | --- | --- | --- | --- | --- | --- | --- |
|  |  | **Chl. a** | **Chl. b** | **T. Chl.** | **Carotenoids** | **Chl. a** | **Chl. b** | **T. Chl.** | **Carotenoids** |
|  |  | **(µg mg^−1^fresh weight)** | | | | **(µg mg^−1^fresh weight)** | | | |
| **Maize** | 0mM | 2.16±0.10**a*** | 1.39±0.02**a*** | 3.55±0.03**a*** | 3.77±0.06**a*** | 2.56±0.01**A*** | 1.58±0.02**A*** | 4.14±0.03**A*** | 5.23±0.02**A*** |
|  | 50mM | 1.97±0.02**b*** | 1.28±0.02**b*** | 3.25±0.04**b*** | 3.63±0.03a***** | 2.20±0.02**B*** | 1.39±0.01**B*** | 3.59±0.03**B*** | 3.92±0.02**B*** |
|  | 100mM | 1.87±0.03**b*** | 1.11±0.01**c*** | 2.98±0.04**c*** | 3.43±0.04**b*** | 2.16±0.02**B*** | 1.25±0.02**C*** | 3.41±0.04**B*** | 3.70±0.01**C*** |
|  | 150mM | 1.54±0.01**c*** | 0.78±0.03**d*** | 2.32±0.04**d*** | 3.08±0.03**c*** | 1.85±0.02**C*** | 1.22±0.01**C*** | 3.07±0.03**C*** | 3.39±0.02**D*** |
|  |  |  |  |  |  |  |  |  |  |
| **Rice** | 0mM | 1.76±0.01**a*** | 1.32±0.02**a*** | 3.08±0.03**a*** | 3.56±0.04**a*** | 2.06±0.02**A*** | 1.40±0.01**A*** | 3.45±0.03**A*** | 4.00±0.01**A*** |
|  | 50mM | 1.70±0.02**a*** | 1.06±0.02**b*ns*** | 2.76±0.04**b*** | 3.50±0.03**a*** | 1.92±0.02**B*** | 1.14±0.02**B*ns*** | 3.05±0.04**B*** | 3.69±0.02**B*** |
|  | 100mM | 1.70±0.01**a*** | 0.78±0.02**c*** | 2.48±0.03**c*** | 3.12±0.02**b*** | 1.53±0.01**C*** | 0.98±0.02**C*** | 2.81±0.03**C*** | 3.67±0.02**B*** |
|  | 150mM | 1.17±0.02**b*** | 0.55±0.01**d*** | 1.72±0.03**d*** | 2.38±0.04**c*** | 1.53±0.02**D*** | 0.89±0.02**D*** | 2.42±0.03**D*** | 3.32±0.04**C*** |
|  |  |  |  |  |  |  |  |  |  |

**Table. S3.** The relationship between physiological and antioxidant parameters versus with and without *Aspergillus terreus* under saline conditions.

| **Maize** (With and without *A. terreus*) | | | | | | | | | | | | | | | | | |
| --- | --- | --- | --- | --- | --- | --- | --- | --- | --- | --- | --- | --- | --- | --- | --- | --- | --- |
|  |  | **Chl_a+b_** | **C _x+c_** | **PI_abs_** | **Fv/Fm** | **qP** | **gs** | **Op** | **Proline** | **H_2_O_2_** | **Phenols** | **MDA** | **CAT** | **SOD** | **APX** | **GPX** |  |
| **RWC** |  | .949** | .858** | .840** | .895** | .842** | 0.942** | .794* | -.749* | -.935** | -0.609 | -.950** | -.749* | -.848** | -0.674 | 0.5 |  |
| **Chl _a+b_** |  |  | .890** | .917** | .945** | .929** | 0.916** | .791* | -0.595 | -.975** | -0.465 | -.893** | -0.666 | -0.672 | -0.553 | 0.475 |  |
| **C _x+c_** |  |  |  | .911** | .834* | .915** | 0.886** | 0.692 | -0.537 | -.821* | -0.393 | -.824* | -0.649 | -0.585 | -0.553 | 0.393 |  |
| **PI_abs_** |  |  |  |  | .897** | .953** | 0.893** | .728* | -0.335 | -.903** | -0.147 | -.776* | -0.438 | -0.443 | -0.33 | 0.592 |  |
| **Fv/Fm** |  |  |  |  |  | .962** | 0.919** | 0.682 | -0.446 | -.969** | -0.363 | -.858** | -0.503 | -0.572 | -0.465 | 0.665 |  |
| **qP** |  |  |  |  |  |  | 0.890** | 0.624 | -0.371 | -.919** | -0.261 | -.820* | -0.491 | -0.461 | -0.436 | 0.606 |  |
| **gs** |  |  |  |  |  |  |  | .812* | -0.574 | -.940** | -0.453 | -.904** | -0.593 | -.709* | -0.537 | 0.54 |  |
| **Op** |  |  |  |  |  |  |  |  | -0.472 | -.781* | -0.32 | -0.62 | -0.434 | -0.614 | -0.241 | 0.298 |  |
| **Proline** |  |  |  |  |  |  |  |  |  | 0.534 | .948** | .794* | .957** | .957** | .927** | 0.035 |  |
| **H_2_O_2_** |  |  |  |  |  |  |  |  |  |  | 0.423 | .895** | 0.58 | 0.647 | 0.502 | -0.555 |  |
| **Phenols** |  |  |  |  |  |  |  |  |  |  |  | 0.7 | .904** | .878** | .926** | 0.157 |  |
| **MDA** |  |  |  |  |  |  |  |  |  |  |  |  | .830* | .842** | .817* | -0.4 |  |
| **CAT** |  |  |  |  |  |  |  |  |  |  |  |  |  | .866** | .954** | 0.091 |  |
| **SOD** |  |  |  |  |  |  |  |  |  |  |  |  |  |  | .841** | -0.195 |  |
| **APX** |  |  |  |  |  |  |  |  |  |  |  |  |  |  |  | 0.031 |  |
| **Rice** (With and without *A. terreus*) | | | | | | | | | | | | | | | | | |
|  |  | **Chl_a+b_** | **C _x+c_** | **PI_abs_** | **Fv/Fm** | **qP** | **gs** | **Op** | **Proline** | **H_2_O_2_** | **Phenols** | **MDA** | **CAT** | **SOD** | **APX** | **GPX** |  |
| **RWC** |  | .948** | .863** | .931** | .925** | .947** | .864** | .804* | -0.628 | -.912** | -0.402 | -.990** | -.802* | -0.661 | -0.61 | -0.447 |  |
| **Chl _a+b_** |  |  | .961** | .906** | .948** | .971** | .911** | .806* | -0.602 | -.985** | -0.345 | -.944** | -.780* | -0.698 | -0.555 | -0.453 |  |
| **C _x+c_** |  |  |  | .811* | .901** | .934** | .868** | .712* | -0.415 | -.946** | -0.142 | -.871** | -0.632 | -0.573 | -0.374 | -0.319 |  |
| **PI_abs_** |  |  |  |  | .959** | .944** | .935** | .806* | -0.537 | -.881** | -0.33 | -.959** | -.824* | -0.571 | -0.634 | -0.408 |  |
| **Fv/Fm** |  |  |  |  |  | .941** | .931** | .749* | -0.438 | -.933** | -0.19 | -.938** | -.719* | -0.511 | -0.495 | -0.268 |  |
| **qP** |  |  |  |  |  |  | .919** | .795* | -0.557 | -.932** | -0.323 | -.972** | -.808* | -0.632 | -0.587 | -0.443 |  |
| **gs** |  |  |  |  |  |  |  | .905** | -0.505 | -.932** | -0.285 | -.892** | -.797* | -0.656 | -0.588 | -0.506 |  |
| **Op** |  |  |  |  |  |  |  |  | -.719* | -.845** | -0.565 | -.804* | -.874** | -.855** | -.747* | -.787* |  |
| **Proline** |  |  |  |  |  |  |  |  |  | 0.595 | .949** | 0.581 | .865** | .915** | .880** | .862** |  |
| **H_2_O_2_** |  |  |  |  |  |  |  |  |  |  | 0.332 | .902** | .756* | .737* | 0.527 | 0.493 |  |
| **Phenols** |  |  |  |  |  |  |  |  |  |  |  | 0.358 | .775* | .791* | .889** | .835** |  |
| **MDA** |  |  |  |  |  |  |  |  |  |  |  |  | .810* | 0.62 | 0.609 | 0.432 |  |
| **CAT** |  |  |  |  |  |  |  |  |  |  |  |  |  | .818* | .947** | .769* |  |
| **SOD** |  |  |  |  |  |  |  |  |  |  |  |  |  |  | .746* | .925** |  |
| **APX** |  |  |  |  |  |  |  |  |  |  |  |  |  |  |  | .773* |  |

Significant level at (p≤0.01) is denoted by using Pearson’s correlation coefficients. ****** Correlation is significant at the 0.01 level (2-tailed).

***** Correlation is significant at the 0.05 level (2-tailed)
